# A termite symbiotic mushroom maximizing sexual activity at growing tips of vegetative hyphae

**DOI:** 10.1186/s40529-017-0191-9

**Published:** 2017-09-19

**Authors:** Huei-Mei Hsieh, Mei-Chu Chung, Pao-Yang Chen, Fei-Man Hsu, Wen-Wei Liao, Ai-Ning Sung, Chun-Ru Lin, Chung-Ju Rachel Wang, Yu-Hsin Kao, Mei-Jane Fang, Chi-Yung Lai, Chieh-Chen Huang, Jyh-Ching Chou, Wen-Neng Chou, Bill Chia-Han Chang, Yu-Ming Ju

**Affiliations:** 1 Institute of Plant and Microbial Biology, Academia Sinica, Taipei, 11529 Taiwan; 2 Graduate Institute of Biotechnology, National Changhua University of Education, Changhua, 50074 Taiwan; 3 Department of Life Sciences, National Chung Hsing University, Taichung, 40227 Taiwan; 4 Department of Natural Resources and Environmental Studies, National Dong Hwa University, Hualien, 97401 Taiwan; 5 National Museum of Natural Science, Taichung, 40453 Taiwan; 6 Yourgene Bioscience, New Taipei, 23863 Taiwan

**Keywords:** Symbiosis, *Odontotermes formosanus*, *Termitomyces* mushroom, Meiotic-like, Basidiomycota

## Abstract

**Background:**

*Termitomyces* mushrooms are mutualistically associated with fungus-growing termites, which are widely considered to cultivate a monogenotypic *Termitomyces* symbiont within a colony. *Termitomyces* cultures isolated directly from termite colonies are heterokaryotic, likely through mating between compatible homokaryons.

**Results:**

After pairing homokaryons carrying different haplotypes at marker gene loci *MIP* and *RCB* from a *Termitomyces* fruiting body associated with *Odontotermes formosanus*, we observed nuclear fusion and division, which greatly resembled meiosis, during each hyphal cell division and conidial formation in the resulting heterokaryons. Surprisingly, nuclei in homokaryons also behaved similarly. To confirm if meiotic-like recombination occurred within mycelia, we constructed whole-genome sequencing libraries from mycelia of two homokaryons and a heterokaryon resulting from mating of the two homokaryons. Obtained reads were aligned to the reference genome of *Termitomyces* sp. *J132* for haplotype reconstruction. After removal of the recombinant haplotypes shared between the heterokaryon and either homokaryons, we inferred that 5.04% of the haplotypes from the heterokaryon were the recombinants resulting from homologous recombination distributed genome-wide. With RNA transcripts of four meiosis-specific genes, including *SPO11*, *DMC1*, *MSH4*, and *MLH1*, detected from a mycelial sample by real-time quantitative PCR, the nuclear behavior in mycelia was reconfirmed meiotic-like.

**Conclusion:**

Unlike other basidiomycetes where sex is largely restricted to basidia, *Termitomyces* maximizes sexuality at somatic stage, resulting in an ever-changing genotype composed of a myriad of coexisting heterogeneous nuclei in a heterokaryon. Somatic meiotic-like recombination may endow *Termitomyces* with agility to cope with termite consumption by maximized genetic variability.

**Electronic supplementary material:**

The online version of this article (doi:10.1186/s40529-017-0191-9) contains supplementary material, which is available to authorized users.

## Background

Fungus-growing termites (Macrotermitinae, Isoptera) have practiced farming with their fungal symbionts of the basidiomycete genus *Termitomyces* R. Heim (Lyophyllaceae, Agaricales) as the cultivars since ca. 24–34 million years ago in the African rainforest (Aanen and Eggleton 2005; Roberts et al. 2016). Decomposing activity on plant matters of *Termitomyces* symbionts appears complemented by that of termite gut microbiota, which was suggested by analyzing annotated draft genomes from *Macrotermes natalensis* (Haviland) and its *Termitomyces* symbiont along with gut metagenomes from individuals of various termite castes (Poulsen et al. 2014). Fungus-growing termites, much as leaf-cutting ants, cultivate only one fungal symbiont in a colony (Aanen et al. 2009; Katoh et al. 2002; Mueller et al. 2005; Shinzato et al. 2005), which is widely accepted as a genotypic monoculture involving only one genotype of a particular *Termitomyces* species. This is in sharp contrast to genotypic polycultures and species polycultures, which are commonly adopted in human agriculture and have widely been perceived as better farming practices than monocultures, because diverse systems can be more productive, stable, and resistant to invasions than less diverse systems (Cook-Patton et al. 2011).

**Fig. 1:**
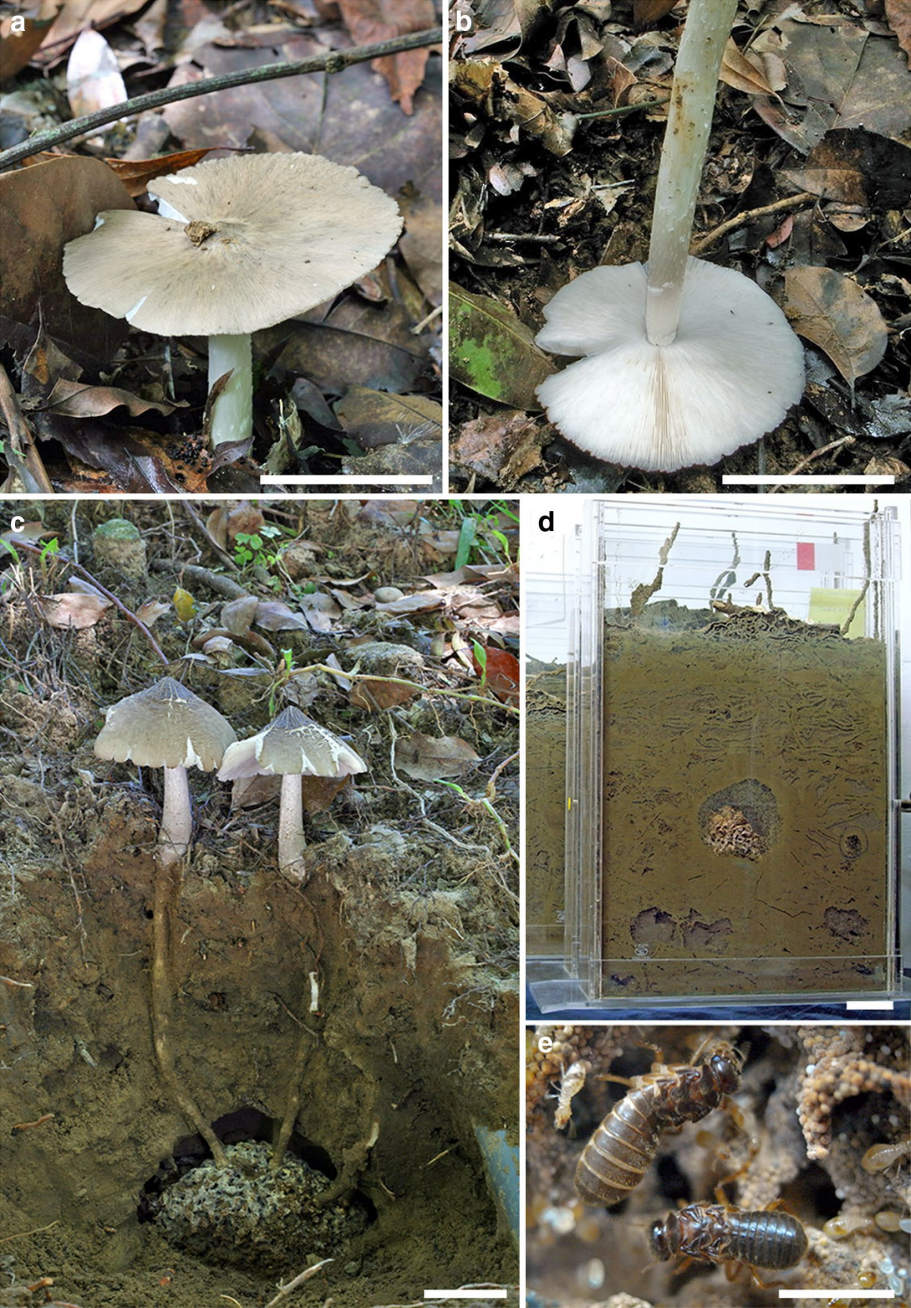
*Termitomyces* mushroom and *Odontotermes formosanus*. **a** Surface of the pileus showing a central umbo. **b** Underside of the pileus showing white gills and a centrally attached stipe. **c** Soil profile to show the fruiting bodies emerging from a subterranean fungus garden. **d** Laboratory-reared termite colony at 3.5 years with a fungus garden ca. 7 cm diam. **e** Queen (*upper*) and king (*lower*) on the fungus garden shown in **d**. *Scale bars* in **a**– **d** = 5 cm, and **e** = 0.5 cm. **a**, **b** From the fruiting body *T0984*; **c** From National Museum of Natural Science, Taichung, Taiwan; and **d**, **e** from a termite colony inoculated with *T0984*

A *Termitomyces* mushroom (Fig. 1a–c) is cultivated by the only macrotermitine species known in Taiwan, black-winged subterranean termite *Odontotermes formosanus* Shiraki (Fig. 1e), at fungus gardens (Fig. 1c, d), which are built underground with fecal balls excreted by termite workers. Two types of spores are produced by the *Termitomyces* mushroom: while conidia are produced at fungus gardens, ingested by termite workers, and repeatedly mixed within fecal balls, basidiospores are only found on the gill surface of fruiting bodies, which emerge above-ground from fungus gardens in summer. Mutualistic relationship between *O*. *formosanus* and its *Termitomyces* symbiont can be demonstrated by rearing of *O*. *formosanus* in laboratory, which only becomes sustainable with incipient termite colonies artificially inoculated with spores of the symbiont (Fig. 1d). Associations between African macrotermitine termites and *Termitomyces* mushrooms have previously been established by artificially inoculating incipient termite colonies with *Termitomyces* spores (Johnson 1981; Johnson et al. 1981; Sands 1960).

**Fig. 2:**
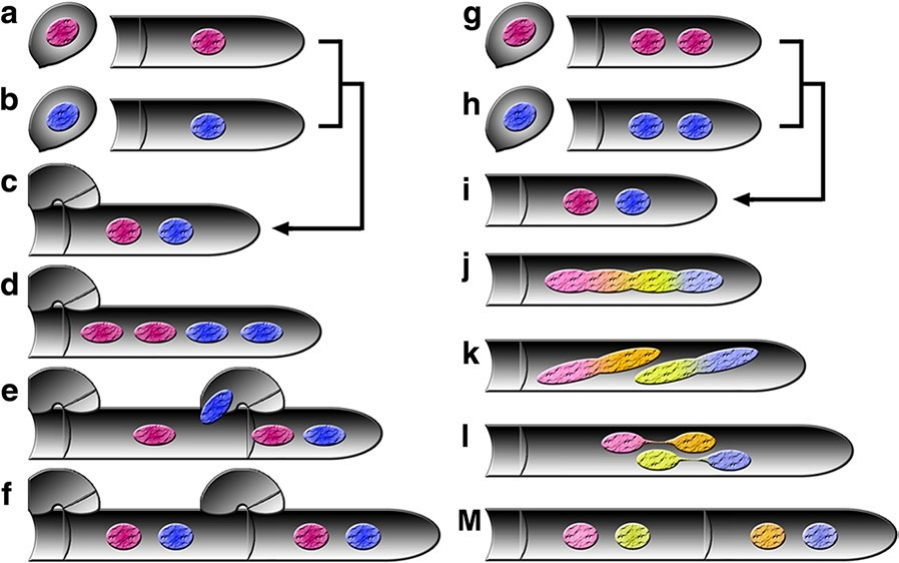
Diagrammatic representation to contrast somatic growths between basidiomycetes with a typical life cycle and the *Termitomyces* symbiont. **a**–**f** Various stages of hyphal growth in a typical basidiomycete, whose heterokaryons (usually dikaryons), unlike those of *Termitomyces*, have a clamp connection at hyphal septa and dikaryotic mycelia, two commonly used indicators for successful matings. **a**, **b** Uninucleate homokaryons carrying different markers (*red* and *blue*) from single uninucleate basidiospores. **c** Binucleate heterokaryon resulting from pairing of two homokaryons. **d** Mitosis giving rise to four nuclei. **e** Formation of clamp connection, through which a blue nucleus migrates back to the penultimate cell, while two new septa are formed in the clamp cell and hyphal tip. **f** The final stage of hyphal cell division. **g**–**m** Various stages of *Termitomyces* hyphal growth. Note that a growing hyphal cell of a homokaryon or heterokaryon is binucleate or trinucleate. Only binucleate tip cells are shown here. **g**, **h** Binucleate homokaryons carrying different markers (*red* and *blue*) from single uninucleate basidiospores. **i** Binucleate heterokaryon resulting from pairing of two homokaryons. Homokaryons and heterokaryons cannot be discerned by nuclear numbers, both having two or three nuclei in a growing hyphal cell. **j** Karyogamy. **k** Stage resembling Meiosis I. **l** Stage resembling Meiosis II. **m** Two cells separated by a newly formed septum as the final stage

A typical life cycle in the majority of basidiomycetes has a prolonged heterokaryotic (often dikaryotic) somatic stage, usually containing two genetically heterogeneous nuclei resulting from fusion of compatible homokaryons (Fig. 2a–f), which is governed by a self-incompatible mating system (Anderson and Kohn 2007; Brown and Casselton 2001; Raper 1966; Raudaskoski and Kothe 2010). Nuclei of heterokaryons stay unfused within every cell until formation of basidia at fruiting bodies, where the nuclei undergo fusion and meiosis (Brown and Casselton 2001). Four basidiospores are normally produced on the top of a basidium, each receiving a nucleus resulting from meiosis. A potentially great genetic variability thus exists among basidiospores, i.e., because they are products of meiosis, where genome-wide recombination can give rise to a great amount of genetic diversity in the offspring.

Here we show that the *Termitomyces* symbiont of *O. formosanus* possesses a life cycle different from other basidiomycetes in having a somatic sexual process involving karyogamy and meiotic-like nuclear division during cell division at active hyphal tips (Fig. 2g–m). The sexual process empowers its growing mycelium a hyperdynamic genotype composed of a myriad of nuclear haplotypes and renders the symbiont with immense genetic variability. The sexual process was confirmed by cytological studies, whole-genome sequencing, and real-time quantitative PCR of four meiosis-specific genes. Pairing tests indicated that mating system of the *Termitomyces* symbiont may not be governed by heterogenic compatibility as in the typical life cycle found in heterothallic basidiomycetes (Fig. 2a–f).

## Methods

### Fungal isolations

*Termitomyces* cultures were isolated from a fruiting body *T0984* collected from Nan-tou County, Taiwan, in 2009. The termite colony was not excavated to confirm its connection with *T0984*. However, by inoculating the conidia produced in culture by *T0984* into colonies of *O. formosanus* (see below), we were able to confirm the symbiotic relationship between the two partners. Two types of fungal cultures were obtained or derived from this fruiting body: a heterokaryon (*FB*) and homokaryons (*BS*). *FB* was isolated directly from the stipe tissue and served as the inoculum for laboratory-reared termite colonies, from which the fungus was reisolated by obtaining single conidia from guts of worker termites from the colonies and was confirmed to be *FB*. The *FB* culture was originally obtained by paring away the stipe surface with a heated razor blade, removing a piece of the stipe tissue from underneath by sterilized forceps, and placing the removed tissue on 2% Difco malt extract agar (MEA, 20 g/L Difco malt extract, 20 g/L Difco agar). Emerging hyphal tips were excised and transferred to fresh MEA. *BS* cultures were obtained from single basidiospores and were used for pairing tests. Those *BS* cultures were initiated from spreading onto MEA a large number of basidiospores, which were collected by placing the pileus of *T0984* on a sterilized plate for several hours, allowing basidiospores to fall from gills. Germination rates of the *Termitomyces* basidiospores were extremely low, and only one out of several hundred started to germinate after 2 days. A fine glass needle was used to gently guide germinating basidiospores bearing a short germ tube to clean areas on the agar, where agar blocks each overlain with a germinating basidiospore were excised and transferred to fresh MEA. We eventually obtained 43 *BS* isolates, whose growth rates varied greatly, 1–4 cm diam/month. Twenty of the *BS* cultures, i.e., *BS02*, *BS03*, *BS04*, *BS05*, *BS06*, *BS09*, *BS10*, *BS11*, *BS14*, *BS17*, *BS18*, *BS19*, *BS21*, *BS26*, *BS27*, *BS31*, *BS32*, *BS35*, *BS38*, and *BS39*, were randomly selected for subsequent pairing tests.

### Termite rearing and fungal inoculation

To test if the *Termitomyces* culture (*FB*) that we isolated from the fruiting body *T0984* was indeed a symbiont of *O*. *formosanus*, laboratory-reared colonies of the termite were initiated from alates captured during nuptial flight on the campus of Academia Sinica, Taiwan in June, 2009. Six dealates were placed in each 9-cm plastic Petri dish containing wet sifted soil to ensure the presence of both sexes. A chamber was created by the dealates at bottom of the soil within a few hours. Eggs were laid in 10 days. After the first brood of workers matured and emerged for foraging ca. 2.5 months after the nuptial flight, conidial suspension (ca. 10^5^ conidia/mL) prepared from the culture *FB* (Table 1) was inoculated into 34 termite colonies, in which 21 formed fungus gardens within several days. They were survived by eight termite colonies after 6 years. The colonies were reared mainly with autoclaved twigs of Norfolk Island pine *Araucaria heterophylla* (Salisb.) Franco.

**Table 1:** Heterokaryons resulting from pairings between 20 homokaryons, which were sorted into four genotypes, i.e., *A*_*1*_ *B*_*1*_, *A*_*2*_ *B*_*2*_, *A*_*1*_ *B*_*2*_, and *A*_*2*_ *B*_*1*_, by haplotypes at *MIP* and *RCB*

	*A*_*1*_ *B*_*1*_ × *A*_*2*_ *B*_*2*_	*A*_*1*_ *B*_*2*_ × *A*_*2*_ *B*_*1*_	*A*_*2*_ *B*_*2*_ × *A*_*2*_ *B*_*1*_ (common at *A*)	*A*_*1*_ *B*_*2*_ × *A*_*2*_ *B*_*2*_ (common at *B*)
Mating pairs	*BS11* × *BS09*, *BS11* × *BS32*, *BS18* × *BS09*, *BS18* × *BS27*, *BS18* × *BS32*, *BS39* × *BS09*, *BS39* × *BS32*	*BS02* × *BS03*, *BS17* × *BS03*, *BS17* × *BS05*, *BS17* × *BS10*	*BS04* × *BS31*, *BS06* × *BS35*, *BS09* × *BS31*	*BS14* × *BS19*, *BS14* × *BS38*, *BS21* × *BS19*, *BS21* × *BS32*, *BS21* × *BS38*, *BS26* × *BS19*, *BS26* × *BS32*, *BS26* × *BS38*
MIP (A)	+	+	ND	+
RCB (B)	+	+	+	ND

For each of the four pairing groups, we randomly selected three homokaryons of a genotype to pair with three homokaryons of another genotype. Note that there were nine mating pairs within each pairing group, but only confirmed heterokaryons are listed

+ Both haplotypes detected, *ND* not determined because of identical haplotype

### DNA extraction, PCR, cloning, and sequencing

All of the cultures that we used in this study were subjected to sequencing of their nuclear ribosomal internal transcribed spacers (*ITS*), mitochondrial intermediate peptidase (*MIP*), and pheromone receptor protein homologue (*RCB*). Mycelia were grown in 100 mL of malt extract broth (20 g/L Difco malt extract) on a rotary shaker at 120 rpm for 6–8 weeks. They were harvested by filtration through Whatman No. 1 filter papers, freeze-dried, and stored at −20 °C. Total DNA from a dried fungal mycelium was extracted by using a SLA-32 automatic nucleic acid extractor (Taiwan Advanced Nanotech Inc.) with a TANBead^®^ Fungi DNA extraction kit. The three nuclear DNA loci, *ITS*, *MIP*, and *RCB* were amplified with the primer pairs *ITS1*/*ITS4* (White et al. 1990), *MAF*/*MAR*, and *MR8F*/*MR8R*, respectively, via polymerase chain reaction (PCR) in 96-well GeneAmpt^®^ PCR System 9700 (Applied Biosystems, Foster City, California, USA) and sequenced with ABI Big-dye primer sequencing kit (Applied Biosystems). The primer pair and PCR condition of *ITS* followed the cited reference (White et al. 1990). See below for the primer pairs and the PCR condition of *MIP* and *RCB*. The resulting sequencing reactions were electrophoresed on an ABI 3730xl DNA sequencer. PCR products were cleaned with PCR-M™ clean-up system (Viogene-Biotek Corp., Hsi-chih, New Taipei City, Taiwan) following the manufacturer’s protocol. While PCR products of homokaryons were sequenced directly, those of heterokaryons were subjected to cloning process using pGEM^®^-T vector System I (Promega, Madison, Wisconsin, USA) or Zero Blunt TOP10 plasmid vector (Invitrogen Inc., Carlsbad, California, USA). The plasmids were extracted with plasmid DNA extraction kit Mini Plus™ (Viogene-Biotek Corp.). Multiple clones of a PCR product were sequenced.

The PCR condition was the same for *MIP* and *RCB*: ca. 100 ng template DNA, 1.0 mM MgCl_2_, 0.2 mM each dNTP, 0.2 μM each primer, and 1.25 U Easy-A High-Fidelity PCR Cloning Enzyme (Agilent Technologies, Santa Clara, California, USA) in a 25 μL reaction. Thermal cycling parameters were: an initial denaturation step at 95 °C for 2 min followed by 30 cycles of denaturing at 95 °C for 40 s, annealing at 57 °C for 30 s, extension at 72 °C for 1 min, and a 7 min final extension at 72 °C. The *MIP* locus used in the current study was a shorter version of that published in (James et al. 2004). The primer pair *MAF*/*MAR* (5′-AATGTGTCTGGTACCCGTTGTCCC-3′/5′-ATGTCCAAACTGGGTCTGGAAAGAT-3′) was designed to frame a 327 bp fragment, where 13 SNP sites were available. The *RCB* locus was amplified with the primer pair *MR8F*/*MR8R* (5′-AACCTGCATACCCTCTCTTCCCTAT-3′/5′-GTAATCCAACGGAAGCACCAAGCAT-3′), which framed a 289 bp fragment with seven SNP sites available. The reverse primer *MR8R* was located within *RCB1* (Aimi et al. 2005), whereas the forward primer *MR8F* was located at a 5′-untranslated region, which was reached by chromosome walking from *RCB1*. The chromosome walking was done with TaKaRa LA PCR InVitro Cloning Kit (TaKaRa Bio Inc., Otsu, Shiga, Japan). Template DNAs were prepared according to the manufacturer’s instructions and were subjected to digestion with *Sal*I. The digested DNA fragments were then ligated with nucleotide linkers and used as templates for two rounds of cassette amplification by PCR, which was conducted with two primer pairs *SU1* (5′-GCATCGTTTGCCCAGACGACAGAA-3′)/*SU2* (5′-ACGCCATGTAGAAACAAGTTCCTG-3′) and *SU7*-*1* (5′-GCGTCGTTCGCCCAGACGACAGAA-3′)/*SU7*-*2* (5′-ACGTCATGTAGAAACAAGTTCCTG-3′). After cassette amplification, all amplified DNA fragments were purified, cloned, and sequenced.

### DNA marker loci for pairings and pairing tests

*MIP* and *RCB* were used as marker loci to confirm whether or not a pairing was successful, because *Termitomyces* species lack phenotypic markers. In other basidiomycetes, *MIP* is closely linked to mating locus *A* (James et al. 2004), whereas *RCB* is located within mating locus *B* (Aimi et al. 2005).

Pairings of the 20 homokaryotic *BS* cultures were conducted for pairs carrying different haplotypes at both *MIP* and *RCB* (*A*_*1*_ *B*_*1*_ × *A*_*2*_ *B*_*2*_ and *A*_*1*_ *B*_*2*_ × *A*_*2*_ *B*_*1*_), at *MIP* only (*A*_*1*_ *B*_*2*_ × *A*_*2*_ *B*_*2*_, i.e., common-B), and at *RCB* only (*A*_*2*_ *B*_*2*_ × *A*_*2*_ *B*_*1*_, i.e., common-A). Genotypes of the 20 homokaryons were determined and sorted into *A*_*1*_ *B*_*1*_, *A*_*2*_ *B*_*2*_, *A*_*1*_ *B*_*2*_, and *A*_*2*_ *B*_*1*_. For each of the four pairing groups shown in Table 1, we randomly selected three homokaryons of a genotype to pair with three homokaryons of another genotype, i.e., nine pairs in each pairing group. Homokaryons of a pair were placed 1 cm apart on a 6-cm petri plate containing MEA. Agar blocks containing hyphae at the confrontation line were isolated after 1 month for molecular typing. For homokaryons of those pairs with different haplotypes at both *MIP* and *RCB*, successful matings would result in heterokaryons within which both haplotypes of *MIP* and both of *RCB* coexist. In those cases where paired homokaryons carrying different haplotypes at only one of the two loci, resulting heterokaryons would be expected to carry either both haplotypes of *MIP* or both of *RCB*.

### Nuclear staining

Nuclei of basidiospores, hyphae, and conidia were stained directly with DAPI (4′,6-diamidino-2-phenylindole in an antifade solution, Vector Laboratories, California, USA). Basidiospores were collected from the fruiting body *T0984*. Hyphae and conidia were obtained from culturing mycelia of *FB*, *BS*s, and paired *BS*s (Table 1) in 250 mL Erlenmeyer flasks, each of which contained a 100 mL liquid medium consisting of 3 g/L Bacto beef extract, 5 g/L Bacto peptone, and 15 g/L d-glucose, and incubated without shaking at 25 °C for 3 week. The stained fungal components were examined by fluorescence microscopy with a Zeiss LSM510 Meta confocal microscope equipped with C-Apochromat 63 ×/1.2 W corr objective. Two channels were used: DAPI channel (specifications: Two Photo Laser Excitation: 740 nm; Transmission 1.5%; Main Beam Splitter: KP650; Beam Splitter 2: mirror; BP 390–415IR; Detector Gain: 650; Amplifier Offset: −0.1) and DIC channel (specifications: Two Photo Laser Excitation: 740 nm; Detector Gain: 130; Amplifier Offset: −0.1).

Hyphae and conidia were also subjected to HCl-Giemsa stains (Giemsa’s azur eosin methylene blue solution, Merck KGaA, Darmstadt, Germany) in accordance with published protocols (Knox-Davies and Dickson 1960; Ward and Ciurysek 1962) with slight modification and is summarized as follows. A harvested mycelium was washed with MQ water, fixed in a freshly prepared Farmer’s fluid (95% ethanol: glacial acetic acid = 3:1) for 1 h, and successively washed in 95 and 70% ethanol, and immersed in MQ water for 10 min. It was then placed in 1 N HCl at room temperature for 10 min, hydrolyzed in 1 N HCl at 60 °C for 8 min, washed in MQ water with 5 changes, suspended in phosphate buffer (0.2% KH_2_PO_4_, 0.4% Na_2_HPO_4_·7H_2_O, pH 7.2) for 5 min, and stained in HCl-Giemsa for 15 min or longer if required. The stained mycelium was washed thoroughly with MQ water and phosphate buffer in series, and was mounted in MQ water. The stained fungal components were examined by light microscopy with a Leica DMRB microscope equipped with a PL Fluotar 100 ×/1.30 oil objective.

Slide culture technique was also used with small blocks (ca. 0.5 × 0.5 cm) of Difco oatmeal agar (OA) (BD, Sparks, Maryland, USA), each of which was inoculated with a culture, sandwiched between a cover slip and a microscope slide, and grown for 2 week. The hyphal mats and conidia that attached on the cover slip were then stained with DAPI and HCl-Giemsa as described above.

### Fluorescence in situ hybridization (FISH)

Hyphae from cultures of the heterokaryon *BS05* × *BS17* and the homokaryon *BS05* were harvested and fixed in freshly prepared Farmer’s fluid overnight. Hyphae were submerged within a drop of 45% acetic acid while being evenly spread onto a microscope slide prior to air-dried. Cell wall digestion was performed with 100 mg/mL lysing enzyme from *Trichoderma harzianum* (Sigma-Aldrich, St. Louis, Missouri, USA) dissolved in PME (50 mM PIPES, pH 6.7; 25 mM EGTA, pH 8; 5 mM MgSO_4_) for 2 h at 37 °C. Probe DNA was prepared with the *ITS1*-*5.8S rDNA*-*ITS2* sequence of the *FB* culture amplified and labelled with digoxigenin-11-dUTP by using PCR DIG Labelling Mix (Roche Diagnostics GmbH, Penzberg, Germany). The PCR condition is as described above for *ITS*. The FISH procedure (Chung et al. 2008) is briefly described as follows. The nuclei on the microscope slide were denatured in 70% deionized formamide in 2× SSC (0.15 M NaCl, 0.015 M sodium citrate, pH 7.5) at 80 °C for 60 s. Hybridization mixture was prepared from 50% deionized formamide, 2× SSC, sheared salmon sperm DNA (1 µg/µL), 10% dextran sulfate, and probe DNA (10–100 ng in 20 µL per microscope slide). Hybridization was performed in a moisture chamber at 37 °C overnight. Hybridization signals were visualized using a rhodamine-conjugated anti-digoxigenin antibody (Roche Diagnostics GmbH). The nuclei were counterstained with DAPI.

For a fungal culture, FISH preparations were conducted for more than ten times, from each of which eight microscope slides were resulted. More than 20 images of a nuclear stage, i.e., prophase, metaphase, anaphase, or telophase, with clear FISH signals were taken from each FISH preparation.

### PCR-free library construction and high throughput sequencing

Genomic DNAs were extracted from dried mycelia of two homokaryons (*BS05* and *BS17*) and a heterokaryon (*BS05* × *BS17*) by using a SLA-32 automatic nucleic acid extractor with a TANBead^®^ Fungi DNA extraction kit. Approximately 1 μg of a genomic DNA was sonicated to ~350 bp with the Covaris M220 system using the following parameters: duty factor 20%, peak/display power 50 W, 200 cycles per burst, 65 s, 20 °C. The sonicated DNA was purified, end-repaired, A-tailed and ligated with adaptors according to the manufacture’s guide (Illumina Truseq DNA PCR-Free LT library prep kit) followed by purification and sequencing. A paired-end DNA-seq library was sequenced using Illumina HiSeq 2500 platform per manufacturer’s instructions. Sequencing was performed up to 250 cycles. Image analysis and base calling were performed with the standard Illumina pipeline. In total 17,621,957, 23,362,228, and 18,348,369 reads were aligned to the reference genome *Termitomyces* sp. *J132* (Poulsen et al. 2014), with the estimated depths of 32.3×, 42.8×, and 33.6× for *BS05* × *BS17*, *BS05*, and *BS17*, respectively.

### Alignments, heterozygous SNP calling, detection of recombinants, and haplotype inference

After the adapter trimming by cutadapt 1.8.1 (Martin 2011), the reads were aligned to the reference genome of *Termitomyces* sp. *J132* (Poulsen et al. 2014), downloaded from http://gigadb.org/dataset/100056, by using Bowtie 2 with local alignment mode to optimize the mapability (Langmead and Salzberg 2012). Alignments of the homokaryon and heterokaryon samples are publicly available at NCBI as *Study SRP073090*.

To identify potential recombinants, we first searched for heterozygous SNPs with two alleles, as a proxy to the parental alleles. During the SNP calling, the alignments with low mapping quality (MAPQ score <10) were skipped. A heterozygous SNP was identified when the following conditions were met: the depth was ≥10 reads, the two major alleles accounted for ≥90% of the reads, and the difference between them was ≤20%. In total we found 71.5, 60.9, and 74.7 K heterozygous SNPs in *BS05* × *BS17*, *BS05*, and *BS17*, respectively. Due to the high variability of fungus genomes, a large number of the SNPs were likely due to the genetic difference between our strains and the reference strain. We further filtered out the SNPs from the heterokaryon that were also detected in either of the two homokaryons.

Reads that had haplotypes phased by at least five heterozygous SNPs were subjected to haplotype inference. The two most frequently observed haplotypes were considered parental haplotypes, whereas other haplotypes were recombinants. These haplotypes are summarized in Table 2 and are listed in the Additional file 1.

**Table 2:** Recombinants in the homokaryons and heterokaryon

	*BS05*		*BS17*		*BS05* × *BS17* ^a^		Filtered *BS05* × *BS17* ^a^	
	Number of reads	Percentage (%)	Number of reads	Percentage (%)	Number of reads	Percentage (%)	Number of reads	Percentage (%)
Non-recombinants (2 haplotype)	1,832,757	80.28	1213,986	80.71	1450,156	81.41	401,623	94.96
Recombinants (30 haplotypes)	450,166	19.72	290,056	19.29	331,170	18.59	21,336	5.04
Total	2282,923	100	1504,042	100	1781,326	100	422,959	100

The table is a summary for haplotypes phased by at least 5 heterozygous SNPs within a read. The 32 (=2^5^) haplotypes, i.e., two non-recombinants and 30 recombinants, were inferred from every 5 heterozygous SNPs on the same reads

^a^The heterokaryon *BS05* × *BS17* resulted from a mating between the two homokaryons *BS05* and *BS17*. The filtered *BS05* × *BS17* was obtained by excluding those recombinant haplotypes of *BS05* × *BS17* that were shared by *BS05* or *BS17* in the haplotype inference (see “Methods” for detail)

**Fig. 3:**
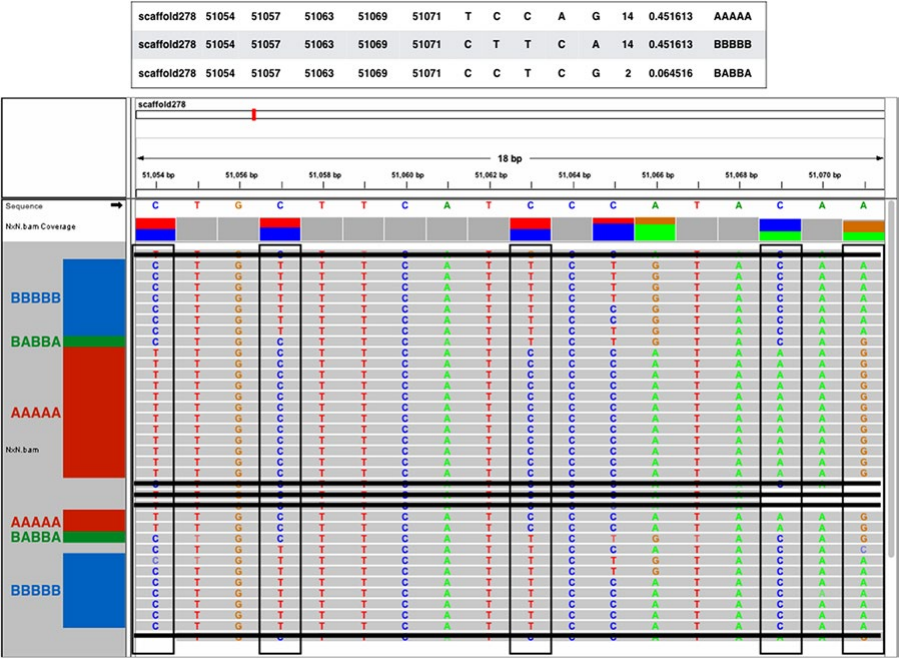
An IGV screenshot showing inference of somatic meiotic-like recombination at scaffold 278

An example of haplotype inference is presented here to demonstrate how we inferred haplotypes from the PCR-free libraries that we constructed for the heterokaryon (*N* × *N*). The screenshot of Integrative Genomics Viewer (IGV) (Robinson et al. 2011) in Fig. 3 shows an alignment of 32 reads at scaffold 278 of the filtered heterokaryon, where 14 reads of one parental haplotype, 14 reads of the other parental haplotype, and two reads of two recombinant haplotypes have been aligned to the reference (indicated by a black arrow). The five heterozygous SNPs at positions 51,054, 51,057, 51,063, 51,069, and 51,071 are framed in black rectangles. Five incomplete reads are crossed out in black lines.

### Reverse transcription PCR (RT-PCR) and real-time quantitative PCR (RT-qPCR)

Total RNA was isolated from a mycelial sample and a gill sample using TRIzol (Invitrogen Inc.) following the manufacturer’s protocol. cDNA synthesis was performed using the First-Strand cDNA Synthesis Kit (Roche Diagnostics GmbH) and anchored-oligo(dT)18 primers. RT-qPCR reactions were performed using FastStart Universal SYBR Green Master (Roche Diagnostics GmbH). Translation elongation factor 1 alpha (*TEF1α*) was used as the internal normalization control with the primer pair GGTGTCAGGCAGCTCATCGT/CGGTCCTCACTCCACTTGGT. PCR was performed on the QuantStudio 12K Flex ABI 7500 real-time PCR system in 8-tube strips. The exon-spanning primer pairs used for RT-qPCR analyses of *SPO11*, *DMC1*, *MSH4*, and *MLH1* are: CCAGCCACGAAACGAGACAT/GCTCAAGAGTGGCAGCGATAT, AAATATTCTCTACGCCCGTGCAT/AAGTCCTTGTCCTCGGCAAA, GCGCCTTGCGAAGATAGAGA/CGATTGCGATGGGCCTTA, and TCTGCACTGAAAGAGCTGGTAGA/GGTCCGATTTGCGTATTCCA, respectively. The RT-qPCR reaction was performed in triplicate for each sample. Specificity of the amplifications and Ct values were analyzed using ABI PRISM dissociation curve analysis software.

## Results

### Pairing tests

The mating system of *Termitomyces* mushrooms has not been verified by pairing studies previously, being inferred heterothallic only in a study of the *Termitomyces* symbiont of *Macrotermes natalensis* from South Africa (de Fine Licht et al. 2005). We set out to pair 20 homokaryons (Table 1), which were obtained from the fruiting body *T0984* produced by a *Termitomyces* symbiont of *O. formosanus* in Taiwan. The molecular markers that we used had two haplotypes (alleles) at each of the two gene loci, mitochondrial intermediate peptidase (*MIP*), which is closely linked to mating type locus *A* in other studied mushrooms (James et al. 2004), and pheromone receptor (**RCB), which is a region within mating type locus *B* in other mushrooms (Aimi et al. 2005; Brown and Casselton 2001). Successful matings would render both haplotypes of *MIP*, denoted as *A*_*1*_ and *A*_*2*_, and both of *RCB*, denoted as *B*_*1*_ and *B*_*2*_, coexisting within a resulting heterokaryon. Cloning and sequencing amplicons of the two loci from the resulting heterokaryons confirmed that the two original haplotypes had come together in a heterokaryon as we expected from successful matings (Table 1). In other basidiomycetes, mating behavior dictated by two loci is considered tetrapolar heterothallism, where compatibility between paired homokaryons requires different haplotypes at each locus, whereas incompatibility occurs when only one or both of the two loci has the same haplotype (Brown and Casselton 2001; Raper 1966). Interestingly, pairings between homokaryons of the *Termitomyces* symbiont that were heterozygous at either *MIP* only (*A*_*1*_ *B*_*2*_ × *A*_*2*_ *B*_*2*_) or *RCB* only (*A*_*2*_ *B*_*2*_ × *A*_*2*_ *B*_*1*_) also resulted in heterokaryons with a normal mycelial growth, suggesting that self-incompatible mating is not strictly followed by or even lacked in the *Termitomyces* symbiont.

### Nuclear behavior revealed by cytological studies

**Fig. 4:**
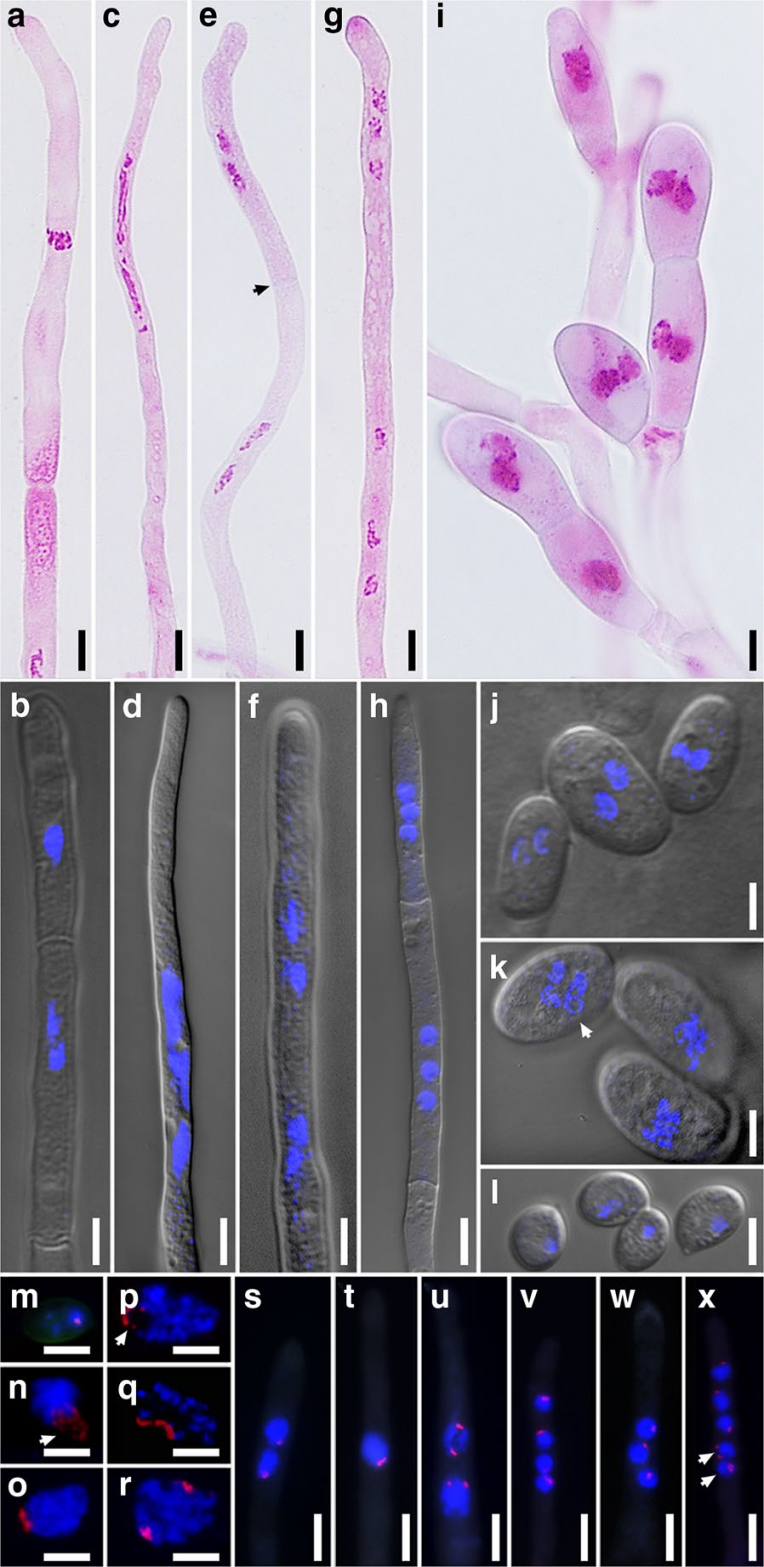
Nuclei at various stages of the *Termitomyces* symbiont. **a**–**h** Vegetative hyphal tips. Nuclei in **a**, **c**, **e**, **g**, and **i** were stained with HCl-Giemsa, whereas those in **b**, **d**, **f**, and **h** were with DAPI. **a**, **b** A fused nucleus in the terminal cell. Nuclei of the penultimate cell in **b** are about to fuse. **c**, **d** Elongated nuclei at the stage resembling Meiosis I. **e**, **f** Nuclei resulting from a binucleate parent cell right after the stage resembling Meiosis II. Septum (*arrowhead*) is forming to bisect the four nuclei in **e**. **g**, **h** Nuclei resulting from a trinucleate parent cell right after the stage resembling meiosis II. **i** Conidiophore attached with developing conidia, each of which has a fused nucleus or two nuclei ready to fuse. **j**, **k** Conidia. Those in **j** are binucleate, whereas those in **k** are either uninucleate or quadrinucleate (*arrowhead*). **l** Uninucleate basidiospores. **m**–**x** Nuclei labelled with *red* FISH probes at *ITS1*-*5.8S rDNA*-*ITS2* region and counterstained with DAPI to render the nuclei *blue*. **m** Haploid nucleus of a basidiospore showing a FISH signal. **n**–**r** Nuclei at various stages of the meiotic-like process released from hyphae. **n** Nucleus at prophase showing FISH signals on four stringy chromatids attached to a nucleolus (the *dark region* pointed by an *arrowhead*). **o** Nucleus at prophase. **p** Nucleus at early metaphase showing FISH signals on four condensed chromatids attached to a nucleolus (the *dark region* pointed by an *arrow*). **q** Nucleus at metaphase, with chromosomes shown as condensed *blue spots*. **r** Nucleus at anaphase. **s**–**x** Nuclei at vegetative hyphal tips, which appear hazy owing to cell wall being partially digested by enzymes. **s** Two haploid nuclei before karyogamy. **t** Fused nucleus at prophase. **u** Two nuclei at a stage resembling anaphase of meiosis II. **v** Four nuclei after a meiotic-like division. **w** Three haploid nuclei before karyogamy. **x** Aberrant hyphal tip showing five postmeiotic nuclei with two (*arrowheads*) still active in division. *Scale bars* in **a**–**m**, **s**–**x** = 5 μm, and **n**–**r** = 2.5 μm

Curiously, cytological studies on heterokaryons resulting from successful pairings indicated that meiotic-like nuclear behavior occurred somatically in *Termitomyces*. Nuclei in a mycelium stained with HCl-Giemsa or DAPI were shown to be in a dikaryotic or trikaryotic arrangement in a newly formed hyphal tip cell (Fig. 4a–h). Nuclei in the cell fused into a single nucleus before dividing into four and six in the dikaryotic and trikaryotic cells, respectively. A septum was then formed to bisect the divided nuclei, resulting in two or three nuclei per cell depending on the nuclear number of their parent cells. The nuclear region distributed with nuclear ribosomal RNA genes (rDNA), which are organized in tandem arrays of multiple copies, was probed by fluorescence in situ hybridization (FISH) (Fig. 4m–x). A chromosome set had one FISH signal only as demonstrated in uninucleate basidiospores (Fig. 4m). Multiple FISH signals were frequently observed in close association within a nucleus, suggesting that pairing of homologous chromosomes indeed occurred (Fig. 4n–q, t). While single FISH signal was normally detected in postmeiotic nuclei at hyphal tips (Fig. 4v), nuclei with two FISH signals (Fig. 4x) or more were occasionally found. The same nuclear behavior was also observed in the culture isolated from stipe tissue of the fruiting body *T0984* (*FB*) and the cultures reisolated from termite colonies inoculated with *FB*.

Cytological studies were also extended to homokaryons derived from single basidiospores, which were uninucleate but germinated into mycelia each containing both dikaryotic and trikaryotic cells. Interestingly, the hyphal cells divided in exactly the same fashion as those of heterokaryons. The sexual process revealed in the homokaryons is similar to the same-sex mating reported from the human pathogenic fungus *Cryptococcus neoformans* (Lin et al. 2005).

Conidia are commonly perceived as asexual spores of fungi, but in the *Termitomyces* symbiont, regardless of whether conidia were produced from heterokaryons or homokaryons, their nuclei, two or three per conidium, underwent karyogamy and a meiotic-like process as those of active hyphal cells (Fig. 4i, k). *Termitomyces* conidia, unlike those of other fungi which serve as clonally propagating and dispersal agents, are produced within nodules at fungus gardens within sequestered termite colonies. Nodules, which contain thin-walled, inflated cells and hyphae in addition to conidia, are consumed by termite workers as an important part of the termite diets (Batra and Batra 1979). Conidia appear to be the only fungal elements surviving the alimentary tracts. Fecal balls excreted and deposited onto fungus gardens by worker termites thus mainly contain conidia and ingested foraged plant debris. Conidia germinate within hours after fecal ball deposition to resume the fungal growth at fungus gardens.

### Occurrence of genetic recombination at somatic stage

**Fig. 5:**
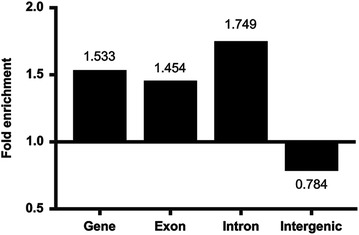
Distribution of recombinants in different genomic regions. The fold enrichment was calculated by comparing the percentage of the recombinants in a specific genomic region against the percentage of the region in the genome

Recombination of homologous nuclear DNA does not normally occur within a lone heterokaryon in other basidiomycetes. To detect if genetic recombination occurred during vegetative growth in the *Termitomyces* symbiont, we performed whole-genome sequencing of two homokaryons *BS05* and *BS17* and a heterokaryon *BS05* × *BS17*, which resulted from mating of the two homokaryons, with an Illumina HiSeq 2500 platform. The sequencing libraries were deliberately constructed without involving PCR amplifications to avoid PCR-mediated recombination (see Additional file 2: Text S1), and obtained reads were aligned to the reference genome *Termitomyces* sp. *J132* (Poulsen et al. 2014). To identify recombinants among the reads, we reconstructed haplotypes by phasing heterozygous SNPs in close proximity. Estimated recombinants from *BS05*, *BS17*, and *BS05* × *BS17* were 19.72, 19.29, and 18.59%, respectively (see Table 2; Additional file 1). The recombinants found in the homokaryons possibly resulted from long-term accumulation of gene duplication and/or repetitive sequences within a genome, which is known to be rich in fungal genomes (Raffaele and Kamoun 2012), or from chromosomal rearrangements (Heitman et al. 2013; Perkins 1997) (see *Possible account for recombination events occurring in the homokaryon* of “Discussion”). We thus excluded the recombinant haplotypes from alignments of *BS05* × *BS17* that were shared with *BS05* or *BS17*. Estimated recombinants in the resulting alignments of the total haplotypes were 5.04% (Table 2). Genetic recombination occurred genome-wide, with a slight, less than twofold enrichment and depletion at genic and intergenic regions, respectively (Fig. 5).

### Expression of meiosis-specific genes

**Fig. 6:**
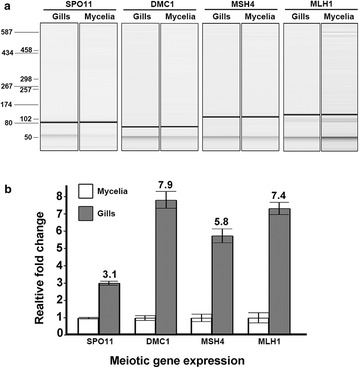
Expression of four meiotic genes in both mycelia and gills. In basidiomycetes, meiosis is commonly considered to be restricted to basidia, which are crowdedly arranged as a palisade-like layer on the surface of gills. **a** The RT-PCR results detected by the high-resolution capillary electrophoresis. The size markers in the RNA ladder on the *left* are in bp. **b** Gene expression determined by RT-qPCR using exon-spanning primers. The expression of translational elongation factor 1 alpha (*TEF1α*) was used as a reference to normalize the data

To further test if the genetic recombination occurring in mycelia is meiotic-like, four meiosis-specific genes, including *SPO11*, *DMC1*, *MSH4*, and *MLH1*, which are not normally expressed at somatic stage in other organisms (Schlecht and Primig 2003), were selected for examination in a mycelial sample. Fruiting bodies of a typical mushroom species have thin, vertically oriented, plate-like gills beneath the cap, which are entirely overlain with crowdedly arranged basidia. Meiosis is commonly considered to be restricted to basidia (Anderson and Kohn 2007; Kües 2000). A gill sample was thus included and compared with the mycelial sample for expression of the four meiosis-specific genes. RNA transcripts of the four genes from the two samples were first detected by reverse transcription PCR (RT-PCR) (Fig. 6a), and the confirmed RNA transcripts were cloned and used for designing primers for real-time quantitative PCR (RT-qPCR) analyses. Results of RT-qPCR showed that all four meiotic genes were expressed in the mycelial sample (Fig. 6b). Although higher expression of these four meiotic genes was detected in the gill sample, successful amplification and <30 threshold cycle value (Ct) in the mycelial sample confirmed their expression in mycelia. The difference in expression levels between mycelia and gills may be due to cells (i.e., basidia) undergoing meiosis in the gills being higher in percentage than those (i.e., only hyphal tips) in the mycelia. While gene expressions do not always correspond to gene functions, it should be noted that meiotic genes, especially genes involving recombination, are normally under tight expression control to prevent deleterious consequences of genomic instability.

## Discussion

### Sexual process in the *Termitomyces* mushroom

The novel life cycle that we present here for the *Termitomyces* symbiont represents a highly interesting case in evolution of sex, where the fungus maximizes the sexual process by vigorously reshuffling its genetic makeup in a meiotic-like fashion during each somatic cell division and conidial production irrespective of whether or not mating has occurred. It is to be found out whether or not this process continues in tissue of the fruiting body and terminates at basidia. Basidia are the only sites of sexual activity known in other basidiomycetes, where sex (karyogamy and meiosis) and reproduction (basidiospore formation) are tightly coupled (Aanen and Hoekstra 2007). Sex of the *Termitomyces* symbiont prevails at somatic stage and looks as if uncoupled with reproduction process except for the brief duration of basidiospore production. Nonetheless, growth of vegetative hyphae in fungus gardens can readily be diverted to conidial production after colonization of fecal balls, and conidia are sexual rather than asexual. The coupling between sex and reproduction in the *Termitomyces* symbiont is not tight, but more frequent than what it appears by way of conidial production.

Sexual processes have been widely regarded as being costlier than asexual ones (Barton and Charlesworth 1998; Bell 1982; Burt 2000; Maynard Smith 1971; Otto 2009; Williams 1975). The “cost of meiosis” or “cost of sex” remains a puzzling question in evolutionary biology despite the fact that sex in general has been accepted by many for generating genetically variable progeny, upon which natural selection can act to accelerate evolution for adaption and for eliminating deleterious mutations. Nests of fungus-growing termites are sequestered environments, where *Termitomyces* fungi are essentially sheltered from outside (Mueller et al. 2005). The nests are generally considered homeostatic with fine-tuned microclimates, which are characterized by high moistures, elevated temperatures ca. 30 °C, and high CO_2_ concentrations, in favor of *Termitomyces* growth (Korb 2003; Thomas 1987; Turner 2004) but stressful to other microbes, which are relatively low in diversities (Cooke and Whipps 1987). If the nest environments are in homeostasis and if sex is for adaptation, what could be the driving force propelling the *Termitomyces* symbiont to keep sexuality over asexuality? Mycelia and nodules of the symbiont are frequently and constantly under termite consumption, which undoubtedly imposes an immediate, continuous pressure upon the symbiont, whose cellular components, notably nuclei and DNA-containing organelles, will be digested if they fail to enter indigestible conidia. Conidia are thus like Noah’s ark in that only a certain number or percentage of cellular components can gain entrance into them to ultimately survive the termite gut passage. Strong competition in entering conidia among rivalling cellular components may exist and exert a direct driving force upon the symbiont to produce conidia high in genetic variability as opposed to clonality in order to adapt to the next round of termite consumption.

### Choice of using FISH in detecting ploidy level of nuclei at hyphal tips

Flow cytometry has been employed extensively in detecting ploidy level of nuclei. However, using this method to study nuclei located at hyphal tips is extremely difficult, if not infeasible, unless hyphal tip cells could be excised and collected selectively to pass through the narrowing channel of a flow cytometer. FISH with rDNA (ITS1-5.8S rDNA-ITS2) as the probe DNA, on the other hand, is a useful method for visualizing and determining ploidy level of nuclei at hyphal tips, where the meiotic-like divisions occur. The images shown in Fig. 4m–x are representatives at various nuclear stages observed during our FISH experiments. For example, the nucleus at Fig. 4n was unmistakably identified as a diploid at prophase because it had four FISH signals on four stringy chromatins, which means a pair of 45S rDNA in a diploid genome undergoing a duplication process at this stage. Nuclei in Fig. 4v–x were haploid resulting from meiotic-like division because there was only one FISH signal detected in each of the nuclei.

### Possible account for recombination events occurring in the homokaryon

Fungal genomes are highly dynamic, rich in chromosomal rearrangement (Heitman et al. 2013; Perkins 1997). In the *Termitomyces* symbiont, recombinants were detected not only in the heterokaryon *BS05* × *BS17* but also in the homokaryon *BS05*. We excluded the possibility that the recombinants in the homokaryon sample originated from contaminants by checking reads at four protein-coding gene loci listed in Additional file 2: Table S2, where only one parental haplotype was detected at each locus.

**Fig. 7:**
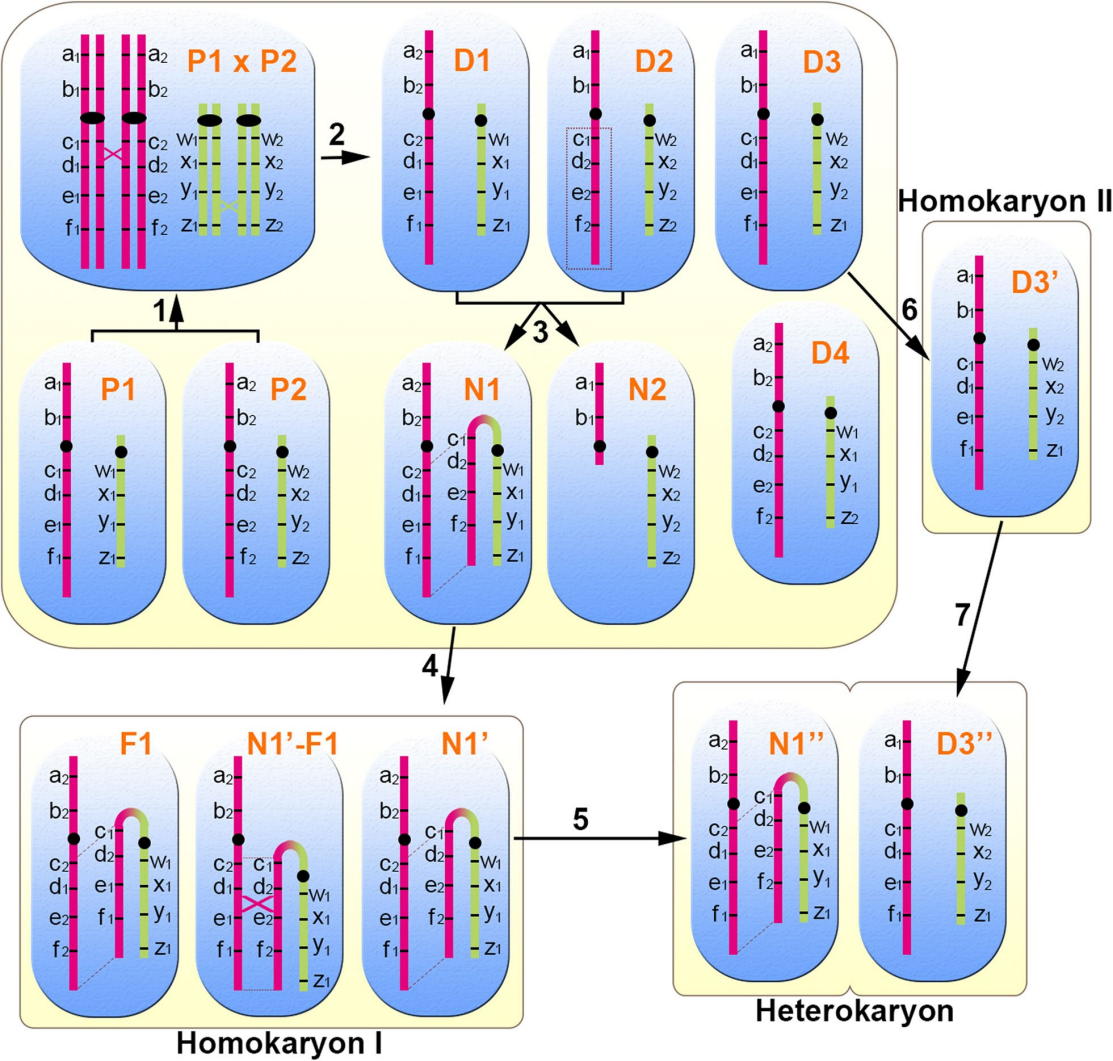
Schematic diagram showing how certain recombinants could have arisen in the heterokaryon but not in the homokaryon and vice versa

To explain how recombinants could have arisen in the homokaryon but not in the heterokaryon and vice versa, we use a schematic diagram (Fig. 7) to demonstrate that one unique recombination was detected in each of the heterokaryon and the homokaryon and that one recombination was shared by both. At step 1, two parental haploid nuclei, P1 and P2, fused to form a diploid nucleus (*P1* × *P2*), with a reciprocal recombination occurring at each of the two chromosome sets, i.e., one between loci c and d, and the other between loci y and z. At step 2, four daughter nuclei, D1, D2, D3, and D4, were produced after the meiotic-like process. At step 3, nuclei D1 and D2 fused, and the chromosome arm carrying alleles c_1_, d_2_, e_2_, f_2_ was translocated onto the chromosome carrying alleles w_1_, x_1_, y_1_, and z_1_. Among the meiotic products, nuclei N1 and N2 require further attention, because N1 acquired the chromosome arm that was lost in N2. At step 4, N1′, a nucleus derived from N1, was encapsulated within a basidiospore, which developed into homokaryon I. Within homokaryon I, F1 was developed from N1′ through the intermediate state N1′–F1, where a non-homologous recombination occurred between loci d and e of the two chromosomes. When homokaryon I mated with homokaryon II, only nucleus N1′’, which derived from N1′, migrated to the heterokaryon (step 5), where it paired with nucleus D3′’, which derived from nucleus D3’ of homokaryon II (step 7). Nucleus D3’ had its origin from nucleus D3 (step 6).

Recombination sites at the heterokaryon that resulted from plasmogamy between homokaryon I and homokaryon II were detected between loci c and d and between loci y and z, while those at homokaryon I were between loci c and d and between loci d and e. In the heterokaryon, the recombination site between loci y and z is considered reliable but the site between loci c and d was filtered out because it was shared by homokaryon I and the heterokaryon. The recombination site between loci d and e was unique to homokaryon I, becoming possible after the chromosome translocation event at step 3.

### Mating system of the *Termitomyces* symbiont not dictated by heterogenic compatibility

Two basic mating systems are known in mushrooms: homothallic (self-fertile) and heterothallic (self-sterile). Only 10% of the mushroom species have a homothallic mating system and can complete a life cycle without mating. The others have a heterothallic mating system and require fusion between mycelia with different mating types, which, depending on mushroom species, are determined by either one locus (bipolar heterothallism) or two loci (tetrapolar heterothallism) (Whitehouse 1949). Compatibility can only be achieved when the two mates have different alleles at each locus, i.e., heterogenic compatibility (Esser 2006).

*MIP* and *RCB*, as initially planned, were used as markers to suggest the mating system of *Termitomyces*. These markers were assumed to be associated with the mating type loci *A* and *B* (Aimi et al. 2005; James et al. 2004), with the mating system of the *Termitomyces* symbiont being assumed to be tetrapolar heterothallism. This assumption was thus made simply because there happened to be exactly two haplotypes for each of the two marker loci among the 45 homokaryons that we obtained from *T0984*. While capability of mating between some homokaryons has been verified in our study, it remains uncertain if mating is indeed a necessity for the *Termitomyces* symbiont to complete a life cycle. Definite answers will be possible only after a reliable fruiting system becomes available. If mating is eventually proven to be required, only heterokaryons will produce fruiting bodies, while homokaryons remain fruitless. Otherwise, both homokaryons and heterokaryons can fruit.

The only previous study concerning the mating system of the *Termitomyces* symbiont of *Macrotermes natalensis* from South Africa (de Fine Licht et al. 2005) suggests that the fungal symbiont is heterothallic, requiring two compatible mating types to complete a life cycle. It is because two haplotypes of internal transcribed spacers (*ITS*) of nuclear ribosomal DNA were detected in cultures obtained from fungus gardens but only one of the two haplotypes was in each of the five single basidiospore cultures that they obtained. Despite the fact that their results agree with data showing that *Termitomyces* cultures isolated from termite colonies are heterokaryotic (Aanen et al. 2009), uncertainty remains because extensive pairings between single basidiospore isolates (homokaryons) were not conducted and, moreover, a reliable system for mushroom fruiting was not developed.

### Somatic genetic recombination known or suspected to exist in other basidiomycetes

In many basidiomycetes, somatic genetic recombination does not normally occur but can be triggered in a heterokaryon upon its confrontation with a homokaryon (the Buller phenomenon) during an incompatible heterokaryon-homokaryon mating, (*A*_*1*_ *B*_*2*_ + *A*_*2*_ *B*_*1*_) × *A*_*1*_ *B*_*1*_. The resulting recombinant *A*_*2*_ *B*_*2*_ nuclei then migrate from the heterokaryon into the homokaryon to pair with *A*_*1*_ *B*_*1*_ nuclei (Anderson and Kohn 2007; Raper 1966). In addition to the Buller phenomenon, somatic genetic recombination in basidiomycetes is known to occur in three different ways: a meiotic-like process, parasexuality, and internuclear specific factor transfer (Anderson and Kohn 2007). A meiotic-like process is more likely for the *Termitomyces* symbiont, because specific factor transfer is highly unlikely to be genome-wide, and parasexuality occurs in extremely low frequencies.

We suspect that the novel life cycle that we describe in this study can also be found in other *Termitomyces* species and in basidiomycetes of other genera. Intragenic recombination has been reported from *EF1a* and other DNA regions among *Termitomyces* isolates from 31 colonies of *Macrotermes natalensis* in South Africa, which apparently belonged to a breeding population but curiously lacked apparent mushroom fruiting (de Fine Licht et al. 2006). Matings between compatible haploid monokaryons in two honey mushrooms, *Armillaria gallica* (Peabody et al. 2005) and *Armillaria tabescens* (Grillo et al. 2000), resulted in a haploid dikaryon and subsequently a persistent diploid monokaryon by karyogamy. Tissue of fruiting bodies, however, contained haploid dikaryotic cells and was shown to be genetically mosaic, implying somatic genetic recombination during haploidization. Somatic genetic recombination were occasionally detected within dikaryons of *Schizophyllum commune* (Frankel 1979; Parag 1962), and episodic somatic recombination was monitored and detected in culture of dikaryons for 18 months (Clark and Anderson 2004).

## Conclusion

Despite the fact that the sexual process that we found in the *Termitomyces* mycelia is much like meiosis, we still have reservation in considering it typical meiosis mainly due to: (1) karyogamy within a hyphal tip cell involving fusion of two nuclei in some cases and three nuclei in other cases, and (2) further confirmation, in addition to our cytological studies, needed on ploidy changes, especially in those cases where three nuclei are involved. Somatic meiotic-like recombination in the *Termitomyces* symbiont is seemingly analogous with VDJ recombination known in human immune system in being able to recombine genetic material somatically. Further studies are needed to investigate whether or not meiotic-like recombination also confers to the *Termitomyces* symbiont similar vigilance and agility to the human immune system in quickly responding to invaders. Our study emphasizes the necessity of further understanding the genetics of *Termitomyces* symbionts, which are organisms centered on the stage of termite agriculture, in order to learn what and how meiotic-like recombination affects the termite agroecosystem. Our findings are likely to generate a profound influence on how we perceive termite agriculture as well as the endurance of *Termitomyces* monoculture with termites over tens of millions of years.

## Additional files

**Additional file 1.** *Termitomyces_haplotypes*.

**Additional file 2.** Additional information.
