# Supplementary material for: A termite symbiotic mushroom maximizing sexual activity at growing tips of vegetative hyphae
Source: Bot Stud. 2017 Sep 19;58:39. doi: 10.1186/s40529-017-0191-9 (PMC5605481; doi:10.1186/s40529-017-0191-9)
Supplement: Supplementary file 2 — Additional file 2. Additional information. [file 40529_2017_191_MOESM2_ESM.docx]

**ADDITIONAL INFORMATION**

**text S1. Reason for constructing sequencing libraries without involving PCR amplification**

PCR-mediated recombination or chimeric formation readily results from PCR amplification of a mixture of highly similar template DNAs (Judo et al. 1998; Smyth et al. 2010). Our earlier attempts to detect recombinants in the heterokaryons were severely interfered with artificial recombinants cropping up during PCR amplification. To demonstrate how our samples were affected by the artificial recombinants, we selected four gene loci, *MIP*, *RCB*, *EF1α* and *RPB2*, and subjected two samples of each gene locus—one with mixed DNAs extracted separately from two homokaryons separately (*N* + *N*) and the other with DNA extracted from a heterokaryon resulting from a mating of the two homokaryons (*N* × *N*)—to PCR amplification and subsequently to high-throughput sequencing with Illumina MiSeq Sequencer (methods detailed below). This test was carried out for three different pairs of homokaryons, *BS02*/*BS03*, *BS05*/*BS17*, and *BS32*/*BS39*, which showed similar percentages of recombinant haplotypes between *N* + *N* and *N* × *N* samples at the four gene loci (Supplementary table S1) and unlikely to separate PCR-mediated recombinants from those recombinants resulting from pairings. In contrast, recombinant haplotypes were not found at *MIP*, *EF1α*, and *RPB2*, but only one out of 535 reads at *RCB* in our PCR-free library of the heterokaryon *BS05* × *BS17* (Supplementary table S2).

*Methods for Conducting High-Throughput Sequencing with Illumina MiSeq Sequencer*.—To amplify the target region, barcoded forward primer 5’-XXXXXXAATGTGTCTGGTAC‌CCGTTGTCCC-3’ and barcoded reverse primer 5’-XXXXXXATGTCCAAACTGGGTCTG‌GAAAGAT-3’ were used for *MIP*, whereas barcoded forward primer 5’-XXXXXXAACCT‌GCATACCCTCTCTTCCCTAT-3’ and barcoded reverse primer 5’-XXXXXXGTAATCCA‌ACGGAAGCACCAAGCAT-3’ were used for *RCB*. “XXXXXX” denotes the barcode region. A 50 μL amplification reaction contained ca. 50 ng template DNA, 0.2 μM of each primer, and 25μL of 2× Phusion PCR master mix (Phusion® High-Fidelity PCR Master Mix with HF Buffer, New England Biolabs, Ipswich, Massachusetts, USA). PCR amplifications were performed with an MJ Mini Thermal Cycler (Bio-Rad Laboratories, Inc., Hercules, California, USA) programmed for an initial denaturation at 95 °C for 2 min, followed by 30 cycles of 95, 57, and 72 °C for 40, 30, and 60 s, respectively, with a 7 min extension at 72 °C as the final step.

The PCR amplicons were electrophoresed on 1.5 % agarose gel with TAE buffer. The expected DNA band (327 bp for *MIP* and 289 bp for *RCB*, 332 bp for *EF1α*, and 290 bp for *RPB2*) was excised and extracted. Amount of a purified product was measured using Quant-iT dsDNA HS assay (Invitrogen Inc.). Amplicons from a set of samples were pooled together in equimolar amount, and an amplicon library was constructed by using TruSeq DNA LT Sample Prep Kit (Illumina Inc., San Diego, California, USA). In brief, the pooled amplicons (500 ng) were treated with end-repair reactions and A-tailing reaction followed by adapter ligations. The adaptor-ligated DNA was amplified with 10 cycles and purified with Agencourt AMPure XP Kit (Beckman Coulter, Inc., Brea, California, USA). After validated by real time PCR, Experion Automated Electrophoresis System (Bio-Rad Laboratories), and Quant-iT dsDNA HS assay, the amplicon library was sequenced using Illumina MiSeq Sequencer with the paired-end 250 program.

**References**

Judo MS, Wedel AB, Wilson C (1998) Stimulation and suppression of PCR-mediated recombination. Nucleic Acids Res 26:1819–1825.

Smyth RP, Schlub TE, Grimm A, Venturi V, Chopra A, Mallal S, Davenport MP, Mak J (2010) Reducing chimera formation during PCR amplification to ensure accurate genotyping. Gene 469:45–51.

table S1. Recombinant rates generated from four gene loci after PCR amplification. Numbers in parentheses are reads of recombinants. “*N* + *N*” denotes mixed DNAs extracted separately from two homokaryons, and “*N* × *N*” denotes DNA extracted from a heterokaryon resulting from a mating of two homokaryons.

|  | *MIP* | |  | *RCB* | |  | *EF1α* | |  | *RPB2* | |
| --- | --- | --- | --- | --- | --- | --- | --- | --- | --- | --- | --- |
|  | *N* + *N* | *N* × *N* |  | *N* + *N* | *N* × *N* |  | *N* + *N* | *N* × *N* |  | *N* + *N* | *N* × *N* |
| *BS02*/*BS03* | 6.9% (8221) | 6.9% (1391) |  | 5.6% (8481) | 5.6% (1509) |  | 6.8% (29247) | 7.0% (10413) |  | 1.3% (5416) | 1.2% (883) |
| *BS05*/*BS17* | 5.3% (3940) | 5.5% (6143) |  | 5.0% (6000) | 4.1% (5946) |  | 5.8% (20435) | 6.8% (17108) |  | 1.1% (3589) | 0.9% (5713) |
| *BS32*/*BS39* | 7.2% (2703) | 7.3% (7960) |  | 6.2% (3745) | 4.6% (6378) |  | 8.1% (23029) | 5.3% (12755) |  | 1.2% (1791) | 1.2% (4486) |

table S2. Reads for four gene loci retrieved from PCR-free libraries of heterokaryon (*BS05* × *BS17*) and homokaryons (*BS05, BS17*). The three numbers separated by slashes are numbers of parental haplotype 1, parental haplotype 2, and recombinants, respectively.

|  | *MIP* | *RCB* | *EF1α* | *RPB2* |
| --- | --- | --- | --- | --- |
| *BS05* × *BS17* | 45/48/0 | 265/269/1 | 142/126/0 | 6/13/0 |
| *BS05* | 135/0/0 | 618/0/0 | 250/0/0 | 10/0/0 |
| *BS17* | 0/72/1^*^ | 0/472/0 | – | 0/12/0 |

^*^The recombinant is considered to be misread.
